# Toward High Performance Anodes for Sodium-Ion Batteries: From Hard Carbons to Anode-Free Systems

**DOI:** 10.1021/acscentsci.3c00301

**Published:** 2023-05-15

**Authors:** Zhaoguo Liu, Ziyang Lu, Shaohua Guo, Quan-Hong Yang, Haoshen Zhou

**Affiliations:** † College of Engineering and Applied Sciences, Jiangsu Key Laboratory of Artificial Functional Materials, National Laboratory of Solid State Microstructures, Collaborative Innovation Center of Advanced Microstructures, Nanjing University, Nanjing, Jiangsu 210093, China; ‡ Shenzhen Research Institute of Nanjing University, Shenzhen, Guangdong 518000, China; § Graduate School of System and Information Engineering University of Tsukuba, 1-1-1, Tennoudai, Tsukuba, Ibaraki 305-8573, Japan; ∥ Energy Technology Research Institute, National Institute of Advanced Industrial Science and Technology (AIST), Central2, 1-1-1 Umezono, Tsukuba, Ibaraki 305-8568, Japan; ⊥ Nanoyang Group, Tianjin Key Laboratory of Advanced Carbon and Electrochemical Energy Storage, and Collaborative Innovation Center of Chemical Science and Engineering (Tianjin), Tianjin University, Tianjin 300072, China

## Abstract

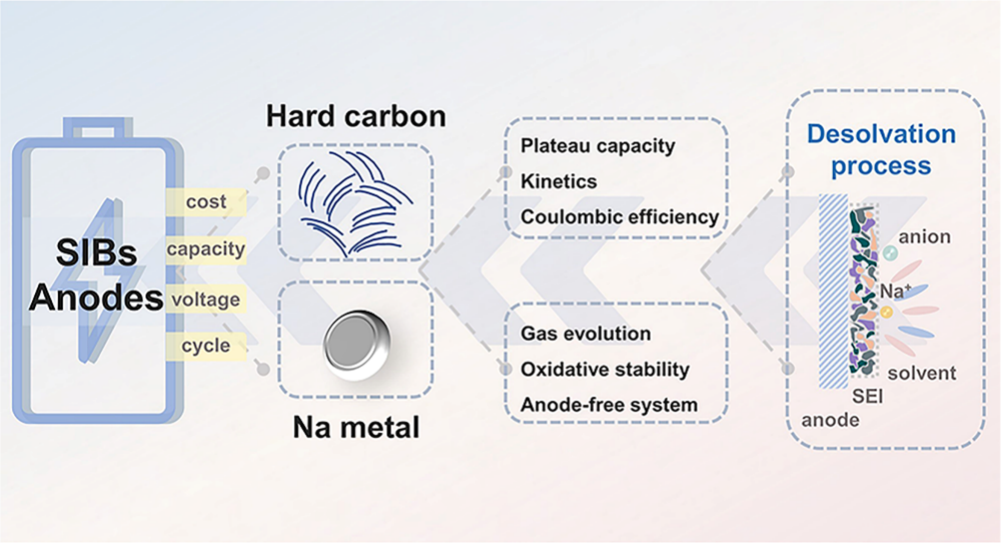

Sodium-ion batteries (SIBs) have been deemed to be a promising energy storage technology in terms of cost-effectiveness and sustainability. However, the electrodes often operate at potentials beyond their thermodynamic equilibrium, thus requiring the formation of interphases for kinetic stabilization. The interfaces of the anode such as typical hard carbons and sodium metals are particularly unstable because of its much lower chemical potential than the electrolyte. This creates more severe challenges for both anode and cathode interfaces when building anode-free cells to achieve higher energy densities. Manipulating the desolvation process through the nanoconfining strategy has been emphasized as an effective strategy to stabilize the interface and has attracted widespread attention. This Outlook provides a comprehensive understanding about the nanopore-based solvation structure regulation strategy and its role in building practical SIBs and anode-free batteries. Finally, guidelines for the design of better electrolytes and suggestions for constructing stable interphases are proposed from the perspective of desolvation or predesolvation.

## Abstract 2

Nanopore-based solvation structure regulation is emerging as an effective strategy to enhance the performance of anode electrodes for sodium-ion batteries.

## 1 Introduction

Compared with lithium (Li), sodium (Na) is more evenly distributed in the Earth’s crust and has much higher reserves, which greatly reduces the cost of sodium-ion batteries (SIBs). Furthermore, the anode of a SIB is compatible with cheap and lightweight aluminum current collectors, which makes SIB technology more sustainable than lithium-ion batteries (LIBs). Therefore, SIBs hold great potential in large-scale energy storage to meet the development of intermittent renewable energy power generation technology.^1^ Cathode materials have exhibited appealing electrochemical performance and acceptable cost, including sodium transitional metal oxides, Prussian blue analogues, and polyanionic compounds.^2−5^ However, the mismatch of the thermodynamic stability between the anode and the electrolyte leads to an extremely unstable interphase whether on typical hard carbon (HC) anodes or Na metal anodes.

**Figure 1:**
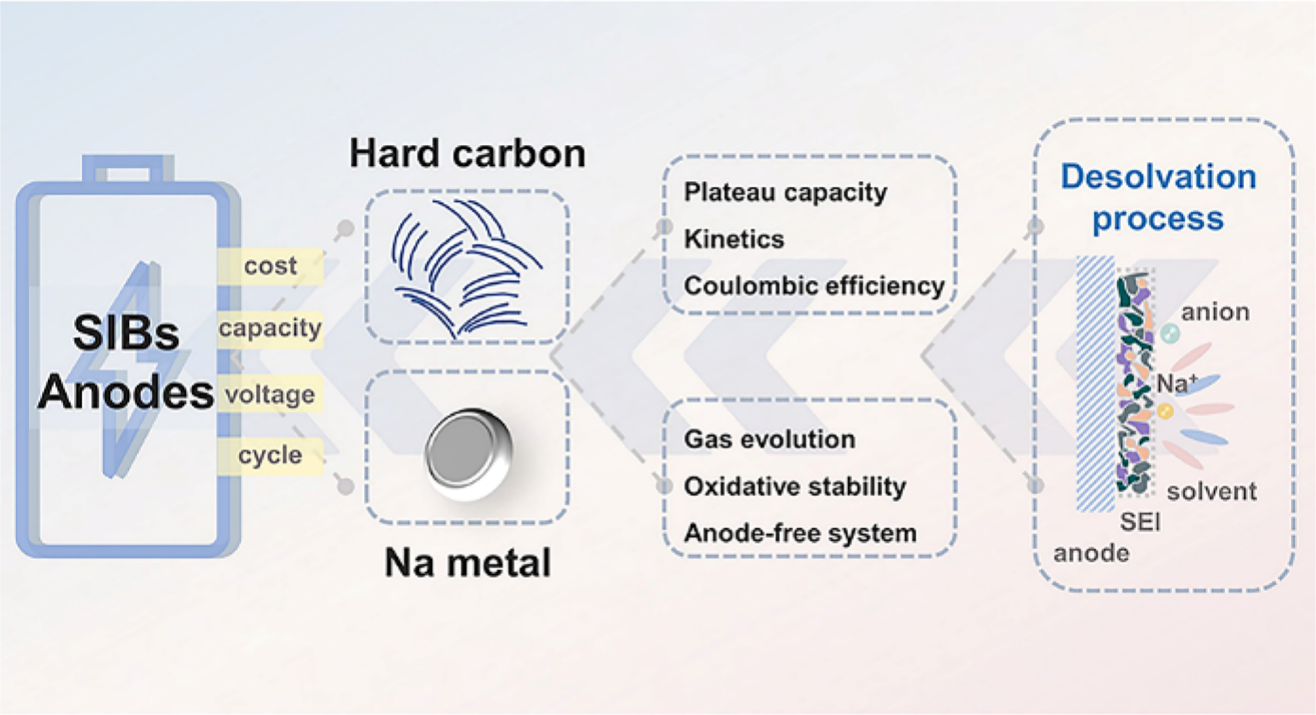
Schematic illustration of regulating the desolvation process to solve the problems of anode electrodes for SIBs.

HC is considered to be the most promising anode material for SIBs due to its low operating voltage, high capacity, and low cost.^6^ However, the long calendar life, rate capability, and initial Coulombic efficiency (ICE) are far from satisfactory. From the prospective of pursuing higher energy densities, the Na metal anode deserves to be refocused due to the high theoretical specific capacity (1166 mA h g^–1^) and low redox potential (−2.714 V versus standard hydrogen electrode potential).^7^ However, its reactivity is higher than that of hard carbon, which would aggravate the electrode–electrolyte interface and result in extremely low CEs in typical ester electrolytes. By building an anode-free battery, its energy density can be fully extracted to make it comparable to state-of-the-art LIBs. While harvesting ultrahigh energy density, it puts forward more stringent requirements for the stability of both cathode and anode interfaces. Obviously, the continued pursuit of high energy density comes with greater challenges. This Outlook comprehensively summarizes the basic scientific issues of anode electrodes from HCs to anode-free systems and generalizes and discusses the nanoconfining strategy in optimizing the desolvation process, as well as its impact on anode interphase stability (Figure 1).

## 2 Hard Carbon Anodes

Different from the stable interaction of Li^+^ in graphite, the intercalation compounds formed by the intercalation of Na^+^ are thermodynamically extremely unstable.^8^ Therefore, the cheap graphite cannot be used as the anode for SIBs. However, HC is composed of randomly oriented and distorted graphene nanosheets that generate randomly distributed graphitized microdomains pores or voids as active sites, which enables it to efficiently store sodium ion (Na^+^).^9^ Considering the narrow layer spacings of other carbon materials and the thermodynamic instability of products, which impeded Na storage, hard carbon materials have attracted general attention as anodes for sodium-ion batteries relying on the outstanding sodium storage performance, various raw materials, and low cost. In addition, owing to its unique advantages including low-cost, high abundance, high capacity, and low redox potential, hard carbon is regarded as the most ideal anode for SIBs.^10^ The Na^+^ storage mechanism in hard carbon is different from the Li^+^ storage mechanism in graphite. Typical discharge curves of hard carbons exhibit distinct slope and plateau regions, which correspond to different Na^+^ storage mechanisms. The slope region is generally attributed to the adsorption of Na^+^ on the surface/defects or the insertion of Na^+^ in few-layer graphene. The plateau region has been assigned to Na clustering in nanopores, which has a vital influence on the discharging capacity and average potential.^11−14^ However, undesirable side reactions between HCs and electrolytes lead to the dissolution and reformation of the solid electrolyte interphase (SEI), resulting in increased impedance and decreased CE. Procedurally, the interfacial electric double layer (IEDL) formed by the adsorption of solvated Na^+^ on negatively charged hard carbon plays a decisive role in interfacial electrochemistry.^15,16^ Hence, the stability of the interface depends not only on the structural properties of hard carbon itself but also on the solvation configuration of the electrolyte.

### 2.1 Accurate Structural Design

In the past few decades, as a conventional strategy to enhance the performance, tuning the structure of HCs has attracted general attention. Precursor reagent screening,^17−20^ optimizing synthesis conditions,^21−23^ gradient design,^14,24,25^ and heteroatom substitution^26−29^ have been adopted to regulate specific characteristics including defect concentration, specific surface area, and the degree of graphitization.^30,31^ The pore and structural features of hard carbon itself are critical for electrolyte–electrode interface stability. Therefore, reconstructing the structural features of pores or voids is proposed as an effective strategy to construct a stable interphase and stabilize the hard carbon anode. The enhanced cycling stability and reaction kinetics of HCs have been achieved by precisely controlling the size of the nanopore. The feasibility of using commercial carbon molecular sieves with well-defined pore sizes as the anode materials of SIBs has been verified, providing a reversible capacity of ∼300 mA h g^–1^ at 100 mA g^–1^ and a high ICE of 73.2%.^32^

**Figure 2:**
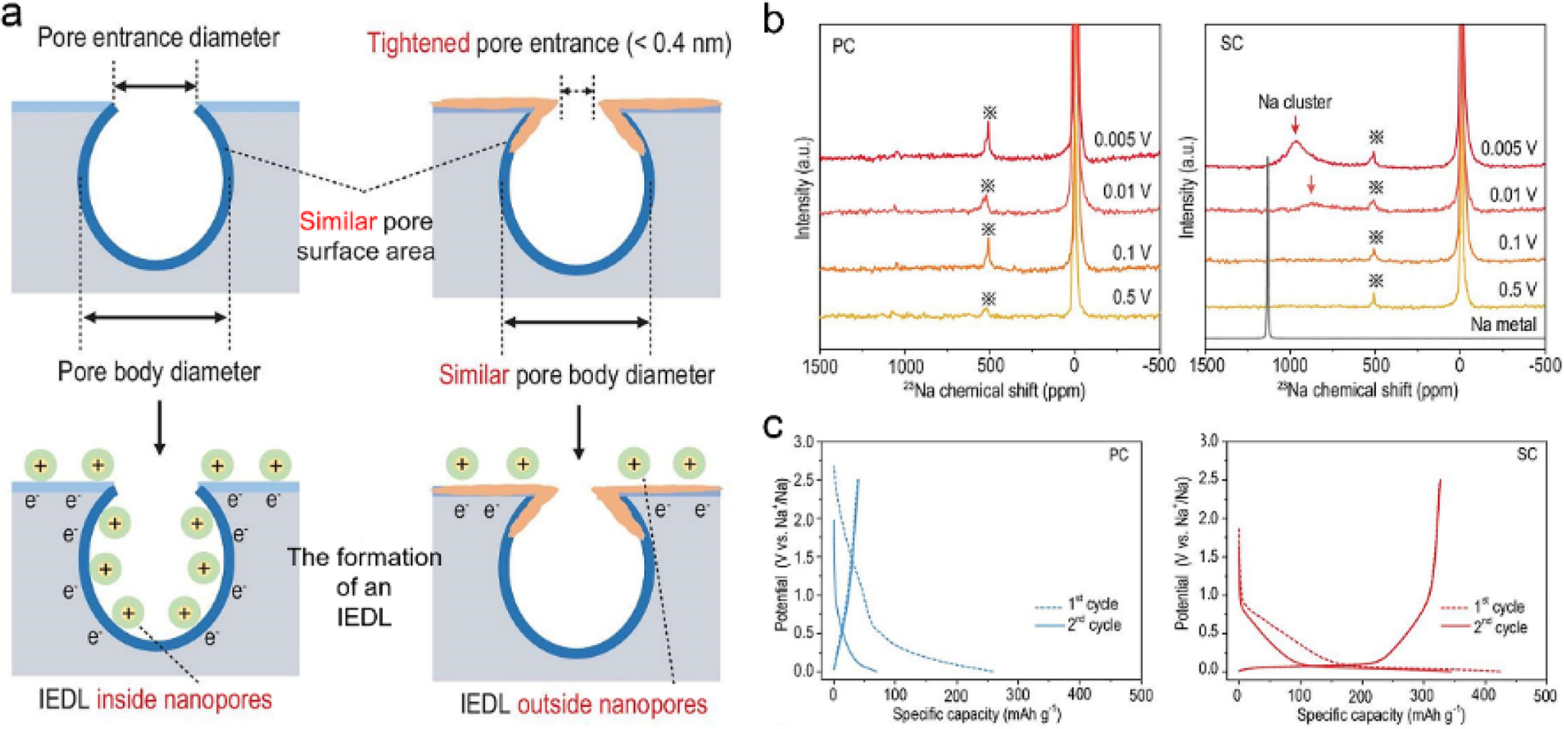
(a) Tightening the pore entrance and the comparison of IEDLs. (b) ^23^Na MAS ssNMR spectra and (c) discharge/charge curves of porous carbon and sieving carbon. Reprinted from ref (16). Copyright 2022 The Authors. Published by Oxford University Press.

Subsequently, the pore entrance size of porous carbon was precisely tightened from micropores (>1 nm) to ultramicropores (0.3–0.5 nm) by Yang and co-workers.^16^ Since the IEDL has a decisive influence on the interfacial electrochemistry and SEI formation, insulating solvent-associated molecules from entering nanopores is essential to promote the formation of a Na cluster in the plateau region (Figure 2a). Chemical vapor deposition (CVD) of methane is used to tighten the pore entrance diameter (PED, < 0.4 nm) and filter out the solvated Na^+^ to shelter the Na clusters by accurate parameter control.^16^ This precisely customized nanopore is accessible to the naked Na^+^ so that the decomposition of electrolytes can be minimized and the interface stability is largely improved, which enables excellent rate and cycling performance as well as a high ICE (80.6%). In addition, the tightened pore entrance and selective body diameter induce suitable Na metallicity and prevent the side reactions between the electrolytes and Na clusters, which have been unraveled by in situ ^23^Na magic-angle-spinning solid-state nuclear magnetic resonance (ssNMR) and operando Raman spectroscopy (Figure 2b). These carefully designed sieving carbons derived from porous carbon provide a plateau capacity over 400 mA h g^–1^ with attractive reversibility (Figure 2c).

**Figure 3:**
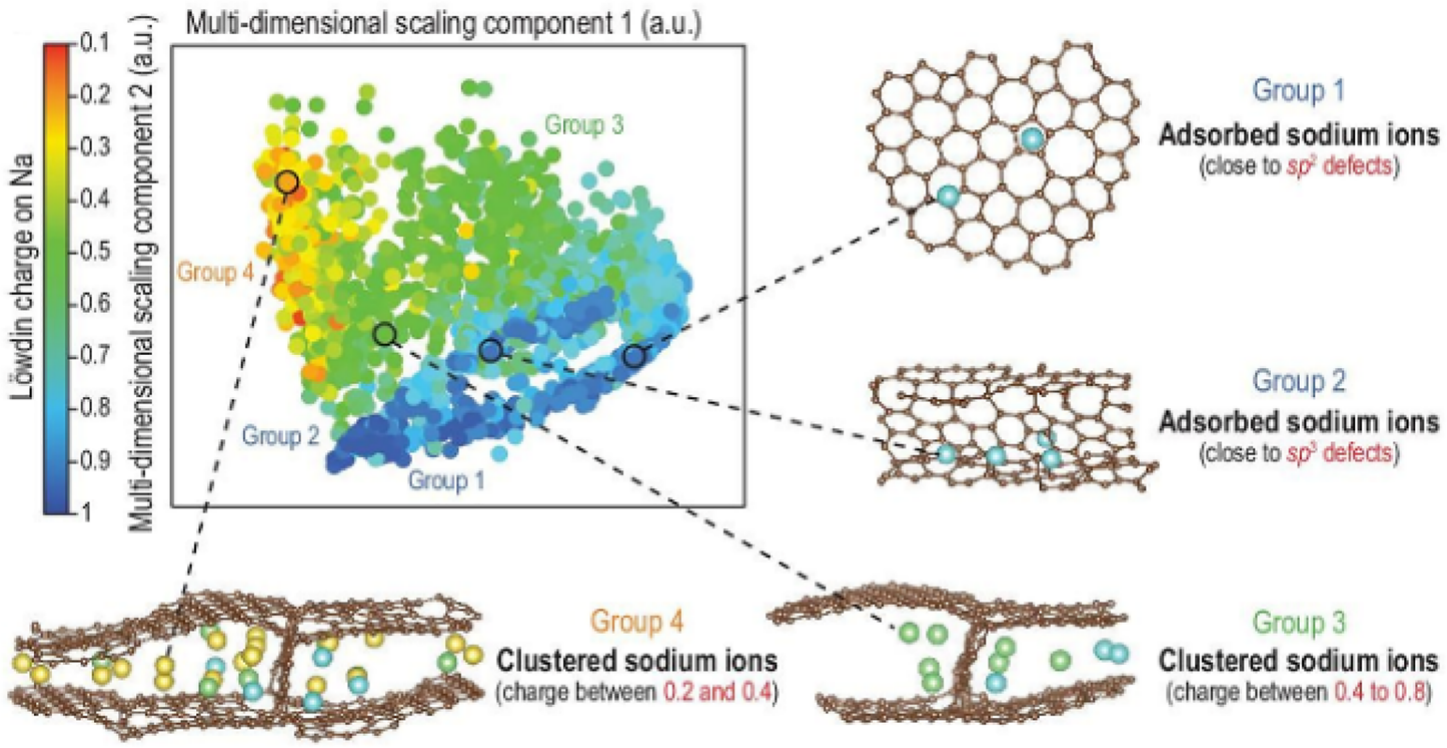
Analysis of the local environment of stored Na^+^ in sieving carbons using a smooth overlap of atomic positions kernel as a structural similarity initially used for Gaussian approximation potential fitting. Reprinted from ref (16). Copyright 2022 The Authors. Published by Oxford University Press.

In addition, machine learning provides insights into dynamic Na cluster formation and the prediction of Na^+^ storage sites in sieving carbons (Figure 3). The simulations show that Na^+^ aggregates and gradually evolves to quasi-metallic Na with increasing Na^+^ insertion, which is very consistent with the results of ssNMR. This work highlights the effect of pore entrance size on Na cluster formation and plateau capacity. The related theoretical simulation, combined with experimental results, provides an invaluable reference for the materials design and mechanism analysis of HCs.

### 2.2 Electrolyte Manipulation

In addition to the intrinsic properties of hard carbons, the compatibility between the electrolyte and the derived SEI has been described as a critical factor that has a significant influence on the charge transfer kinetics.^33−36^ This seriously jeopardizes battery performance, especially the rate capability. Many efforts have been made to address this issue, such as raw material screening,^37,38^ heteroatom doping,^27,39^ defect tuning,^40^ and porous structure optimization.^12^ Unfortunately, the majority of these works have obtained certain improvements at the expense of the low potential plateau (LPP) capacity decreasing. The amount of defects and specific surface areas are negatively correlated with the ICE but positively correlated with the reversible capacity.^41^ The dilemma of the electrochemical capability enhancement of HCs has not been comprehensively resolved by material modification.

**Figure 4:**
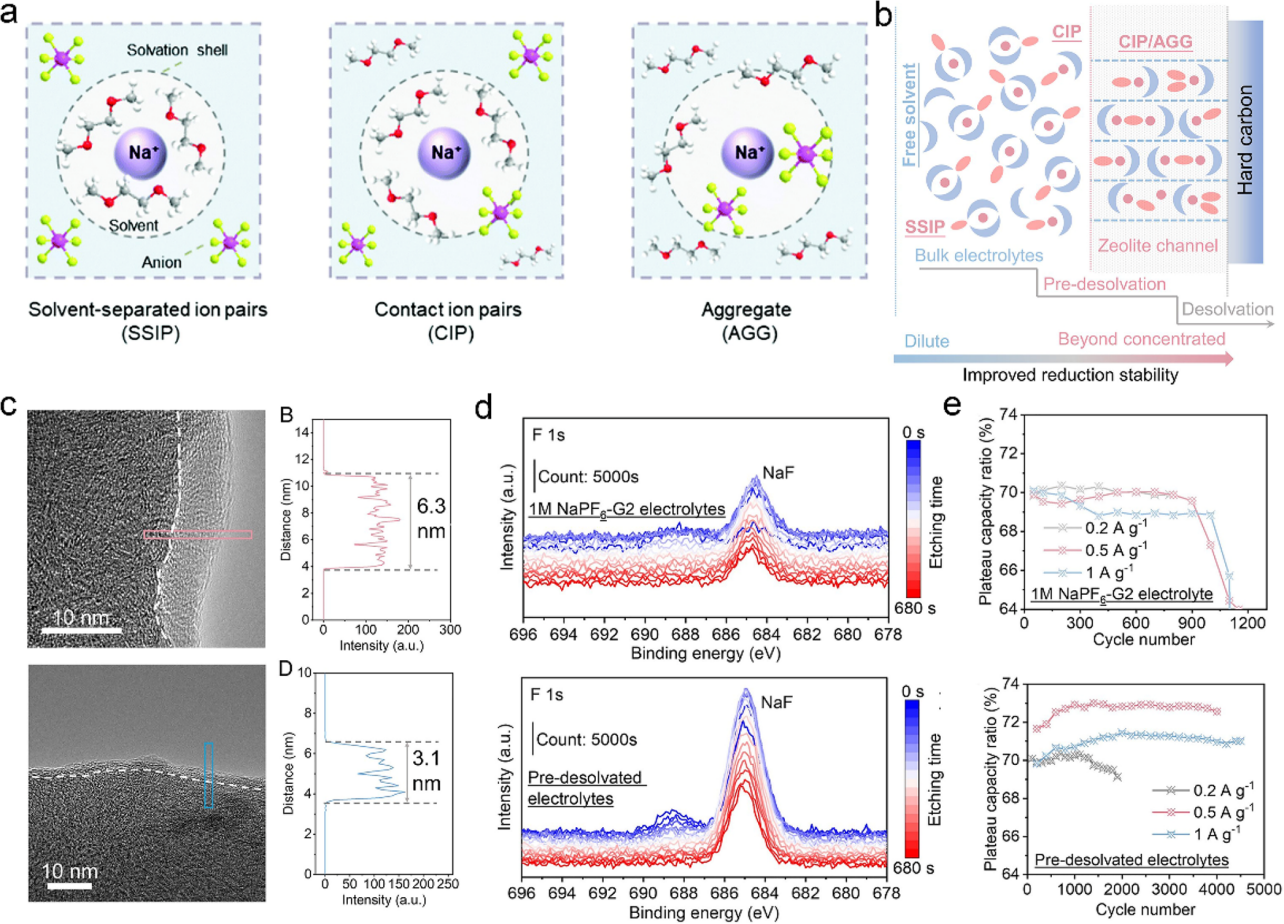
(a) Schematic diagram of the SSIP, CIP, and AGG.^61^. Reproduced with permission from ref (61). Copyright 2022 Royal Society of Chemistry. (b) Schematic diagram of the step-by-step desolvation process. Comparison of (c) the TEM image of hard carbon after 100 cycles, (d) Na fluoride (NaF) XPS signal changes, and (e) the variation of plateau capacity ratios of the pristine ether electrolytes and predesolvated electrolytes.^50^ Reproduced from ref (50). Copyright 2022 The Authors. Published by PNAS.

The theory of the solvation process can be summarized into three aspects, including the dissolution and solvation of Na^+^, the migration of ionic groups, and the desolvation process.^42^ Solvation structures usually include solvent-separated ion pairs (SSIPs), contact ion pairs (CIPs), and aggregates (AGGs) as solvates according to cation–anion interactions (Figure 4a). There are substantial efforts that have been made in solvation structure regulation to optimize the stability of the interphase.^43^ For example, sodium difluoro(oxalate)borate (NaODFB) was synthesized and used in combination with dimethoxyethane (DME), which exhibits enhanced performance at high temperature by forming a special SEI component containing B–F and B–O inorganic groups.^44^ Furthermore, the high-concentration electrolytes bring many benefits, such as anticorrosion perperties, cation transference, interfacial chemistries, and so on, relying on manufacturing solvation sheath structures.^45−47^ Improved performance is also achieved by improving the SEI stability and uniformity using a weakly solvated electrolyte.^48,49^ It is considered that the desolvation process presents a decisive effect on the Na^+^ storage kinetics and the characteristics of SEIs.^50−53^ In regard to the current status, more attention needs to be given to probe the rationale underneath the desolvation process.

Relying on the size effect and particular influence on the solvation structure, porous materials such as metal–organic frameworks (MOFs)^62^ and zeolite molecular sieves (ZMS)^63,64^ have been used to regulate the properties of electrolytes and exhibit unusual performances. Recently, a 3 Å ZMS film is introduced to transform the one-step desolvation process to the step-by-step desolvation process (Figure 4b).^50^ Predesolvation by molecular sieves transforms SSIP-dominated solutions into highly aggregated electrolytes dominated by CIPs and AGGs. This extraordinary stepwise desolvation process adequately dispersed and reduced the high energy barriers of both the desolvation and the activation of Na^+^ transport through the SEI. Additionally, a thin and inorganic-component-rich SEI was generated using the predesolvated electrolytes (Figure 4c). The high abundance of NaF in the SEI results in a very high modulus, which effectively guarantees the mechanical stability of the interphase (Figure 4d). Thereby, the life span and rate capability are dramatically improved, which is much better than using other strategies such as precursor reagent screening, optimization of synthesis conditions, gradient design, and heteroatom substitution (Table 1). What stands out is that the LPP capacity is well maintained with increased current densities, and a LPP capacity ratio of 73% can be maintained at a current density of 0.5 A g^–1^ (Figure 4e). It is worth noticing that this strategy shows excellent versatility, as the electrochemical performance of HC anodes was enhanced in both ester and ether electrolytes. It is generally believed that in ester electrolytes bare Na^+^ is inserted into hard carbon after desolvation.^65,66^ However, in ether electrolytes Na^+^ and the solvated shell coinserted into hard carbons, which has faster reaction kinetics and a lower activation energy for desolvation.^54,61^ This is an important reason why hard carbon anodes have better performance in ether electrolytes. The optimization of the desolvation process provides a novel perspective for facilitating the application of HC anodes.

**Table 1:** Comparison of the Stability and Cycle Number of Hard Carbon Anodes Using Different Strategies

electrolytes	current density (A/g)	cycle number	decay rate (%)	ref
Zeolite film modified	0.2	1900	0.0068	(50)
	0.5	4000	0.0015	
	1	4500	0.002	
HCM-1300-ZBE	2	3000	0.002	(12)
CMS	0.1	300	0.0372	(17)
CPs	0.5	500	0.02	(18)
BPC-11	2	3000	0.0133	(19)
S-HC-p	1	4000	0.005	(29)
HCNS	1	3000	0.0094	(54)
MV-HC	0.2	1100	0.015	(40)
HC-ether	1	2000	0.005	(55)
HCP	0.2	1000	0.007	(56)
HC-DME-0.5%VC	1	2000	0.0022	(57)
HC-0.5 M NaBPh4/DME	1	1500	0.012	(58)
HC-Al2O3	1	2000	0.00655	(59)
HTCS-1000	1	1000	0.0197	(60)

## 3 Na Metal Anodes

### 3.1 Electrolyte Desolvation

#### 3.1.1 Ester-Based Electrolyte

**Figure 5:**
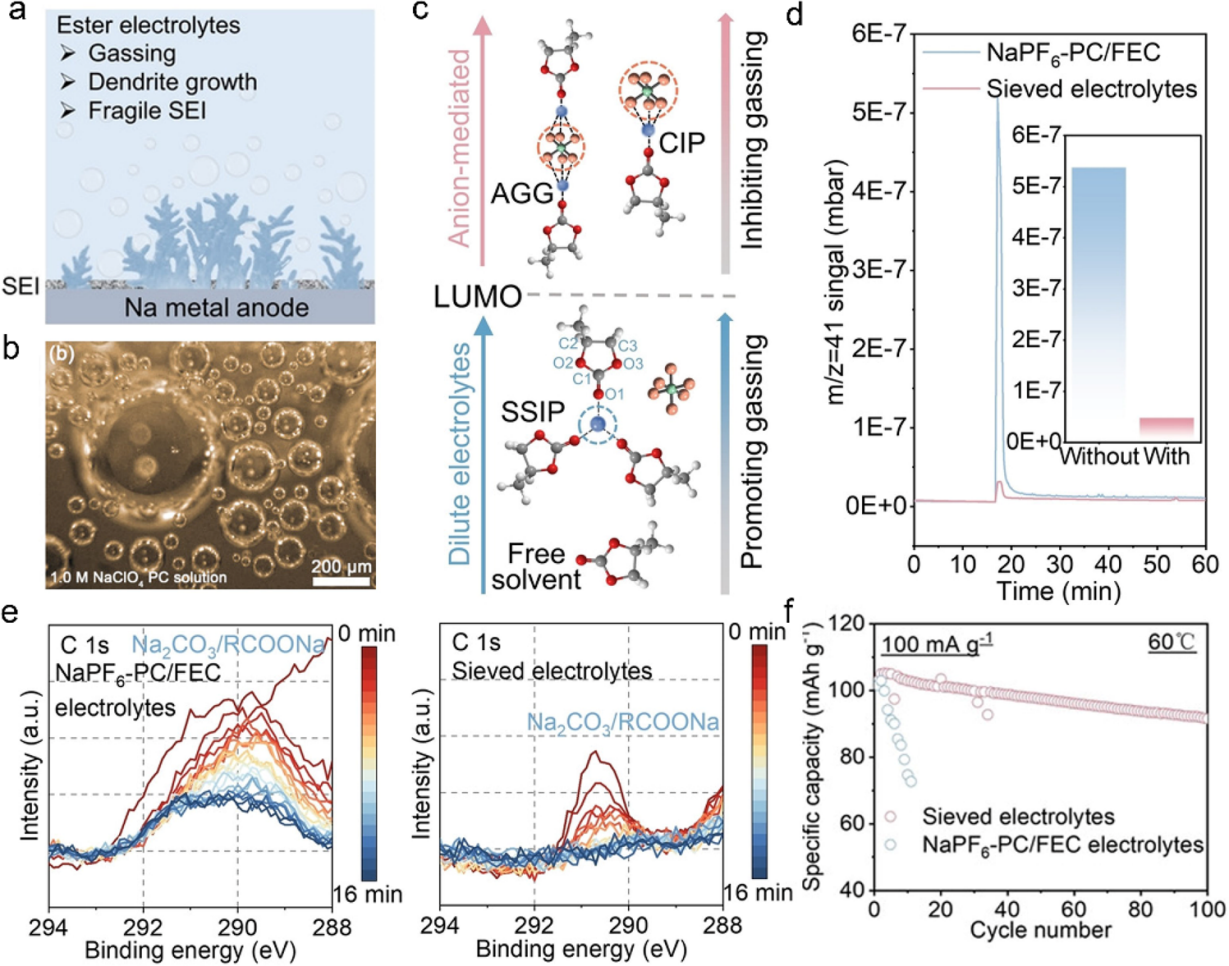
(a) Schematic showing of gassing-related byproducts^83^. Reproduced with permission from ref (83). Copyright 2022 Wiley-VCH. (b) In situ optical microscopic images of gas evolution of Na metal in PC-based electrolytes.^71^ Reproduced with permission from ref (71). Copyright 2017 Wiley-VCH. (c) Relationship between solvation structures and gassing reactivity. (d) Mass signal of C_3_H_6_ (*m*/*z* = 41) after being immersed in Na. (e) Na_2_CO_3_ XPS signal depth profiles of the SEI formed. (f) Cycling stability of Na||NVPF cells at 60 °C in pristine 1 M sodium hexafluorophosphate (NaPF_6_) in propylene carbonate (PC) as the solvent with 5% fluoroethylene carbonate (FEC) additive (NaPF_6_–PC/FEC) electrolytes and sieved electrolytes.^83^ Reproduced with permission from ref (83). Copyright 2022 Wiley-VCH.

In response to the requirements of high energy density for the next generation of Na-based batteries, Na metal anodes hold great promise due to their high theoretical specific capacity and low redox potential. The typical carbonate ester electrolytes generally have excellent antioxidative stability to match a high voltage cathode over 4.0 V. However, there is a strong chemical reactivity between Na metal and the ester electrolyte, leading to persistent parasitic reactions (Figure 5a).^67−70^ When Na metal is in contact with the ester electrolyte, an obvious gas production reaction spontaneously occurs to release combustible gases (H_2_ and alkanes, Figure 5b)^71,72^ that would severely affect the stability of the Na metal anode, leading to extremely poor reversibility and a low CE. Unfavorable solid byproducts further facilitate the formation of fragile SEI and performance degradation.

Constructing protective layers on the surface of Na metal has been demonstrated to be effective.^73−80^ For example, Yu and co-workers advocate that building a sodium bromide (NaBr) coating by the Wurtz reaction or the decomposition of a 1,2-dibromobenzene additive on the Na metal anode effectively reduces the interfacial ion-transport activation energy and restricts Na dendrite formation.^81^ Besides, tuning the solvation structure via some additives, for example, 4-acetylpyridine, has been proposed as an effective strategy.^82^ However, tailoring the desolvation process to stabilize the interface and suppress detrimental gas evolution has not been paid enough attention.

Generally, the solvent molecules show a reduced the lowest unoccupied molecular orbital (LUMO) after complexing with Na^+^, which promotes the reductive decomposition of the electrolyte and leads to gas evolution and unfavorable SEIs.^71^ Manipulating the solvent structure toward anion-mediated CIPs and AGGs can effectively increase the LUMO, thereby reducing side reactions and subsequent gassing (Figure 5c). This has been demonstrated as an effective strategy in high-concentrated electrolytes.^84−86^ However, considering the cost of adding an additional dose of expensive sodium salt, it is of great significance to develop effective and economical methods to facilitate the regulation of the solvation structure. 3 Å ZMS film has been added into the assembly of Na metal batteries (SMBs) and placed between the Na metal and the separator manufacturing sieved electrolytes.^83^ Relying on the size effect, the cation-dominated SSIP is blocked and CIP/AGG is successfully retained in the nanopore due to size effect, which is considered to be similar to superconcentrated electrolytes.^63,87^ The significantly reduced electrolyte activity effectively suppresses the violent decomposition of metallic Na and the release of combustible gases (Figure 5d). In addition, a robust SEI with abundant NaF and Na_2_O was identified, which has been shown to be beneficial for reversible Na plating and stripping. Carbonate-related byproducts are also efficiently suppressed, as demonstrated by X-ray photoelectron spectroscopy (XPS) (Figure 5e).^83^ Finally, Na metal batteries with sieved electrolytes exhibited excellent electrochemical performance even under harsh conditions of high temperature (60 °C) (Figure 5f). This approach provides a new perspective for improving the performance of high-voltage Na metal batteries with ester-based electrolytes.

#### 3.1.2 Ether-Based Electrolyte

**Figure 6:**
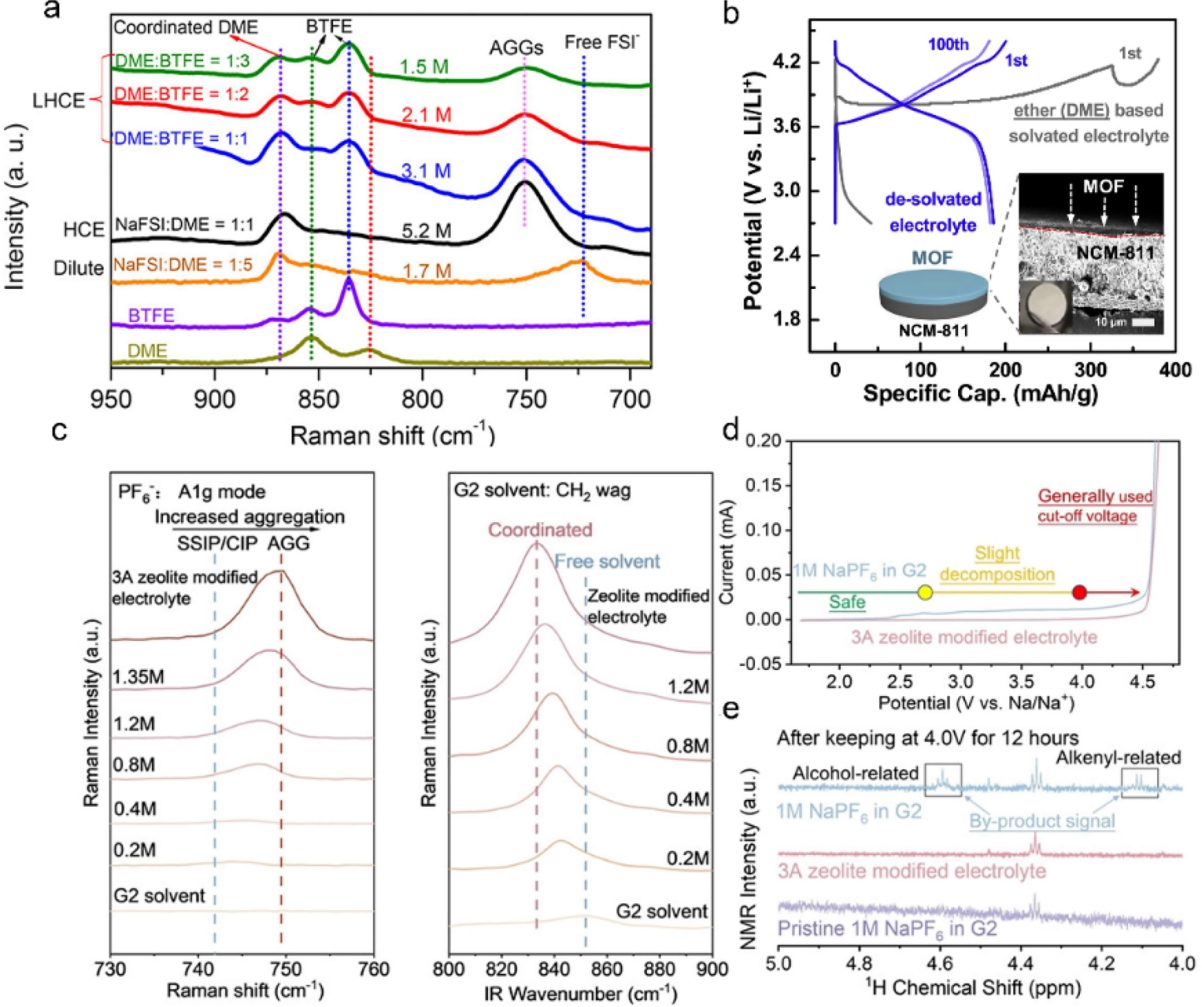
(a) Raman spectra of high-concentration electrolytes and localized high-concentration electrolytes with different sodium bis(fluorosulfonyl)imide (NaFSI) concentrations.^98^ Reproduced with permission from ref (98). Copyright 2018 American Chemical Society. (b) Discharge/charge curves of the NCM-811//Li batteries using typical “Li^+^ solvated ether-based electrolytes” (1 M lithium bis(trifluoromethanesulfonyl)imide (LiTFSI) in 1,3-dioxolane (DOL) and dimethoxyethane (DME), LiTFSI–DOL–DME) and the “Li^+^ de-solvated electrolyte” (1 M Li^+^ desolvated LiTFSI–DOL–DME).^94^ Reproduced with permission from ref (94). Copyright 2020 Elsevier. (c) Raman and FT-IR spectra of various electrolytes. (d) Linear sweep cyclic voltammetry (LSV) tests and (e) ^1^H NMR results of pristine 1 M NaPF_6_–G2 electrolytes and 3A zeolite-modified electrolytes.^99^ Reproduced with permission from ref (99). Copyright 2022 Wiley-VCH.

Ether-based electrolytes have much better compatibility with Na metal anodes due to their excellent antireduction stability.^88^ Nevertheless, the oxidation stability of ether-based electrolytes is far from satisfactory. Adjusting the solvation structure and composition of electrolytes by increasing the concentration has been shown to be effective to reduce the activity of the electrolyte and extend the operating voltage window.^86,89−91^ A localized high-concentration electrolyte was constructed by Zhang et al. by introducing an inert diluent, which exhibited a high CE of 99% (Figure 6a). However, due to the low solubility of salts in ether solvents, it is crucial to explore additives, cosolvents, or other methods to tailor the solvation structure. Some cation additives have been explored and added to the electrolyte to modify the solvation structure and further enhance the electrolyte stability.^92,93^ Recently, MOFs have been used to construct desolvated electrolytes for LIBs (Figure 6b), showing a promising effect in stabilizing a high-voltage cathode in ether electrolytes.^62,94−97^ Therefore, adjusting the solvation structure via sieving effects holds great potential in enhancing the oxidative stability of ether-based electrolytes.

Applying the nanoconfining strategy, Lu et al. and co-workers achieved the regulation of the solvation structure by utilizing the molecular sieves with unique nanopore structures and showed dramatically improved oxidative stability.^99^ Relying on the special size effect, highly aggregate solvation structures (CIP and AGG) are stored in the nanopore, which was verified by the comparison of the Raman and Fourier-transform infrared spectroscopy (FT-IR) signals of different concentrated and zeolite modified electrolytes (Figure 6c). The pristine 1 M NaPF_6_–diglyme (G2) electrolytes undergo obvious decomposition, while the oxidation current of the modified electrolyte is negligible (Figure 6d). The oxidation byproducts such as alcohol-related and alkenyl-related species can be detected in 1 M NaPF_6_–G2 electrolytes (Figure 6e). However, these signals are absent in zeolite-modified electrolytes. Thereby, the half cells of Na||Na_3_V_2_(PO_4_)_2_F_3_ (NVPF) that used this modified electrolyte exhibited an impressive lifespan of more than 800 cycles with a broad voltage window up 2–4.25 V. This nanoconfining strategy by adjusting the solvation structure dramatically improves the oxidation stability ether-based electrolytes.

For all-solid-state electrolytes without applied organic electrolytes, it ensures high safety performance of the Na metal batteries. However, the space charge layer, the poor electrochemical/chemical interfacial stability, and the mechanical compatibility restrain the practical applications.^100,101^ Furthermore, the generally used solid-state electrolytes such as sulfide-based^102,103^ and polymer solid electrolytes^104^ with high ion conductivity are unstable under high voltage. If these problems cannot be properly solved, it will be difficult to achieve the practical application of solid-state Na batteries.

### 3.2 Anode-Free Na Batteries

**Figure 7:**
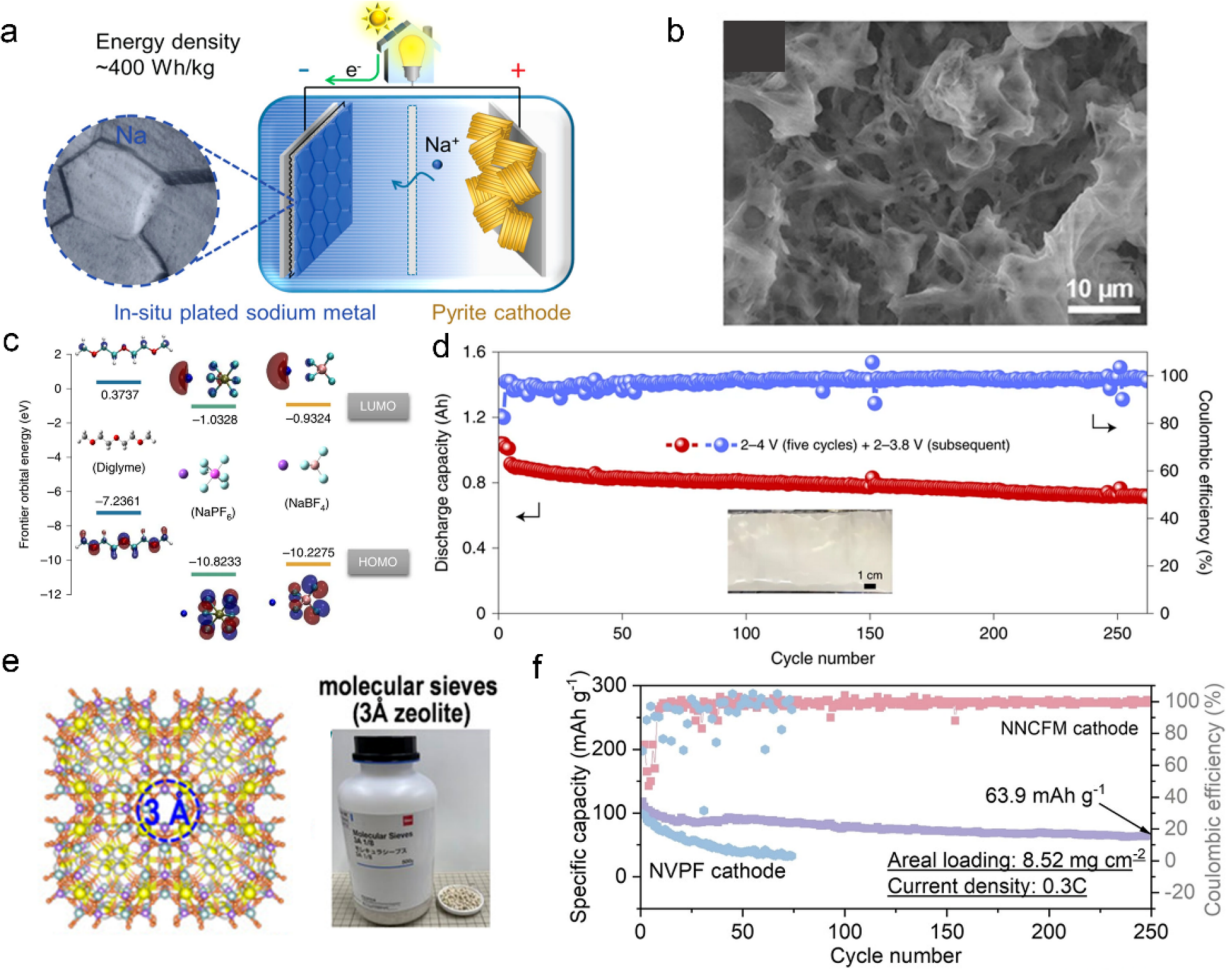
(a) Schematic illustration of an anode-free Na battery system.^112^ Reproduced with permission from ref (112). Copyright 2017 American Chemical Society. (b) Top-view SEM images of the deposited Na on Cu foil using 1 M sodium perchlorate (NaClO_4_) in ethylene carbonate (EC)/diethyl carbonate (DEC) + 5% FEC electrolytes.^113^ Reproduced with permission from ref (113). Copyright 2022 Wiley-VCH. (c) LUMO and HOMO energies of NaPF_6_, sodium tetrafluoroborate (NaBF_4_), and diglyme. (d) Cyclic capability of the Ah-level cylindrical cell.^111^ Reproduced with permission from ref (111). Copyright 2022 Nature Publishing Group. (e) Molecular sieve with 3 Å pore windows.^87^ Reproduced with permission from ref (87). Copyright 2021 Wiley-VCH. (f) The cyclic stability of anode-free Al-NaCu_1/9_Ni_2/9_Fe_1/3_Mn_1/3_O_2_ (NNCFM) full cells and Al–Na_3_V_2_(PO_4_)_2_F_3_ (NVPF) full cells with 3A zeolite-modified electrolytes.^99^ Reproduced with permission from ref (99). Copyright 2022 Wiley-VCH.

With the pursuit of ultimately improving mass and volume energy density, anode-free Na batteries have attracted general attention due to their good economics and facile assembly procedure (Figure 7a). The generally used ester-based electrolytes shows outstanding oxidation stability to match a high-voltage cathode. However, the severe side reactions between Na metal and ester electrolytes and related gas evolution prevent the formation of a stable SEI, resulting in extremely low CE. Therefore, it is inapplicable in anode-free Na batteries (Figure 7b).^105−107^ On the contrary, ether-based electrolytes have been reported to provide a high CE of over 99.9%.^88^ The high reversibility of the Na metal anode in ether electrolytes can be attributed to two aspects. First, the ether electrolyte can generate a NaF- and Na_2_O-dominated SEI, which effectively prevents the penetration of electrolyte and promotes the uniform Na metal deposition. Besides, the unique solvation structure for ether electrolytes enables a much lower activation energy for desolvation, which is favorable for the fast transport of Na^+^ and guarantees the uniform deposition of Na metal.^35,108,109^

By using a 1 M NaBF_4_–tetraglyme electrolyte, a reversible and smooth Na plating/stripping process is achieved, and the constructed Na_2_Fe_2_(CN)_6_//Cu batteries show 76% capacity retention after 100 cycles.^110^ Incorporating a graphitic carbon-coated current collector with a modified ether electrolyte (Figure 7c), Li et al. further increased the cycle life of the anode-free battery to 260 cycles at a charging cutoff voltage of 3.8 V (Figure 7d).^111^ However, the cycling stability is severely compromised when the cutoff voltage is increase to 4.0 V. The current collector modification combined with the optimized electrolyte ensures the high reversibility of the Na anode. In this case, it is actually the high-voltage instability of the ether electrolyte that limits the lifespan.

Therefore, even using the-state-of -the-art ether electrolytes with an advanced current collector strategy such as the three-dimensional structural design of collectors^114−116^ and the artificial sodiophilic layers,^117−122^ the life span of an anode-free battery is generally limited within 100 cycles. Using highly concentrated electrolytes can improve the oxidation stability, and the CE will be greatly reduced at the same time.^94,123^ In fact, as long as ether electrolytes are introduced, the oxidation stability would be an important factor limiting battery lifespan. Lu et al. significantly improved the antioxidation stability of the ether electrolyte by introducing a 3 Å ZMS (Figure 7e) film on the cathode, while the high CE of the Na anode was perfectly preserved. The Al–NVPF anode-free full cells operated stably at a cutoff voltage of 4.25 V. In addition, the Al||NaCu_1/9_Ni_2/9_Fe_1/3_Mn_1/3_O_2_ (Al–NNCFM) anode-free full cells delivered an immense energy density (2–4 V, 369 W h kg^–1^) and a prolonged cycle life of 250 cycles (Figure 7f).^99^ Tailoring the solvation structure of electrolytes by nanoconfining has shown its great potential for the development of anode-free Na batteries.

As discussed above, there are two main factors affecting the life of an anode-free battery. First, the ultrahigh energy density relying on anode-free batteries needs the support of the high operating voltage. The high voltage stability of ether-based electrolytes may be enhanced by constructing dual or multisalt electrolytes or using fluorinated solvents, among others. Besides, even with a high CE of 99.9%, it preserves 36.8% of the pristine capacity after 1000 cycles, which is far from meeting the needs of practical applications. Using carbon materials or sodiophilic materials to promote the uniform nucleation and deposition of Na metal is promising to further increase the CE.

## 4 Conclusion and Outlook

SIBs are expected to play an important role in the development of distributed energy storage systems by virtue of their low cost and abundant raw materials. The stability of the anode–electrolyte interface has been a major stumbling block to the practicality of Na-based cells. We emphasize the size regulation of nanopores in hard carbon anodes and the role of nanoconfined electrolyte regulation in improving the interphase stability. Accurately tailoring the structure of hard carbons and tuning the desolvation process for porous materials are crucial in maintaining the LPP capacity. Furthermore, the nanoconfining strategy shows unexpected performance in inhibiting the gas evolution and oxidative decomposition. Although the nanoconfining strategy has achieved feasible effects, the fundamental mechanism and deeply comprehension have not been unraveled. Therefore, more attention should be paid to the following aspects.

### 4.1 Accurately Tailoring the Structure of HCs

Adjusting the desolvation process has shown tremendous potential in improving or maintaining the plateau capacity of HC anodes. It is crucial to establish the quantitative relation between the LPP capacity and the size of pores. The optimal sodiophilic pores or voids in HCs need to be explored to reach the limit of the LPP capacity. In addition, customizing electrolytes compatible with HCs is a feasible approach to address the stability of HC anodes.

### 4.2 Untangling the Mechanism of the Nanoconfining Strategy

The amelioration in inhibiting gassing and oxidative decomposition has been emphasized through the manipulation of the desolvation process by employing specific porous materials. The driving force for the solvated ions passing through the nonconducting films and related desolvation or predesolvation has not been identified. The interaction mechanism between nanoporous materials and solvated ions remains to be elucidated and verified. Besides, cracks of the film and gaps between particles are inevitable. In situ coating on the surface or cold rolling to fabricate the assisted films may be feasible methods.

### 4.3 Stepping Forward to Anode-Free Systems

The operating voltage of generally used cathodes such as layered oxides, Prussian blue analogues, and polyanion-type cathode are usually not lower than 4 V. Therefore, the focus of building an anode-free battery is the high voltage stability of the electrolyte. Under the premise of maintaining the high CE of the Na anode, the focus in designing a new type of electrolyte is to improve the high voltage stability. In addition, a CE of 99.9% is far from satisfactory for constructing stable anode-free cells. The integration of multiple strategies such as electrolyte optimization and current collector modification is an inevitable trend in the construction of practical anode-free batteries.
